# Etiological Yield of Global Developmental Delay in Kanti Children's Hospital

**DOI:** 10.31729/jnma.3734

**Published:** 2018-10-31

**Authors:** Anshu Jha

**Affiliations:** 1 Department of Pediatrics, Kanti Children's Hospital, Kathmandu, Nepal

**Keywords:** *asphyxia*, *etiology*, *global developmental delay*

## Abstract

**Introduction:**

Global developmental delay is the common pediatric problem having spectrum of underlying causes. Etiological diagnosis is very vital for providing information regarding pathogenesis, prognosis, recurrence, risk and treatment options. The aim of this study was to determine etiological yield of global developmental delay.

**Methods:**

This descriptive cross-sectional study included children of 6 months to 5 year of age with global developmental delay referred to pediatric outpatient Neurology clinic of Kanti Children's Hospital. Diagnostic study included detailed history, examination followed by required investigations neuroimaging, electroencephalogram, hearing and visual assessment. Thyroid function test, karyotyping and enzyme essay were done in selected patients depending on the condition.

**Results:**

In this study, 110 patients were evaluated out of which 70 were male and 40 were female. An etiological diagnosis was determined in 86 (78%) of the patients classified under following categories perinatal asphyxia 49 (44.5%), post infectious sequelae 11 (10%), cerebral dysgenesis 6 (5.45%), genetic syndrome 6 (5.45%), metabolic causes 5 (4.54%), neurocutaneous syndrome 4 (3.63%) and non-specific leucodystrophy changes. Etiology was unknown in 24 (21 %) of the patients.

**Conclusions:**

A specific etiology can be determined in majority of cases of global developmental delay after comprehensive evaluation. The most common etiologies were perinatal asphyxia and post infectious sequelae.

## INTRODUCTION

Global developmental delay (GDD) is a subset of developmental disabilities and defined as a significant delay in two or more domains gross/fine motor, cognition, speech, language, personal/social or activities of daily living.^1^ GDD is one of the most common reasons for referral to pediatric neurology.^2^

The prevalence of GDD is not known precisely however it is estimated to be between 1% to 3%.^3^ Determination of underlying cause for child's delay is of great help in estimation of the child's ultimate development potential. Etiological determination has specific implications regarding treatment, associated condition, estimation of recurrence risk and the design of prevention program. Etiological yield varies ranging from 10% to 80%.^3^ Although, there are several published guidelines still optimal approach remains unclear. Not much data is available in our part of the world regarding GDD.

This study was undertaken to determine etiological yield amongst children of GDD in Kanti Children's hospital and to help to decide investigation and plan of management based on causes.

## METHODS

This is hospital based descriptive cross-sectional study conducted in Kanti Children's Hospital, Maharajganj, Kathmandu, Nepal from August 2017 to December 2017. All patients with global developmental delay of age six months to five years presenting to outpatient neurology clinic of Kanti Children Hospital were evaluated over a period of five months. Ethical clearance was obtained from Institutional Review Board of hospital. Children of age more than 5 years, those in whom only one field of development was affected and children with neurodegenerative diseases were excluded from the study.

Sample size was calculated as following: Sample size (n)= z^2^ x p (1-p)/e^2^ Where

z= 1.96 at Confidence Interval of 95%
P= prevalence 3%^3^
e= error 5%, which came out to be 45. So, Sample size was 45.

All patients coming to outpatient department meeting the inclusion criteria over five months were included. Convenient sampling was done. Incomplete history by parents in case of home deliveries and lack of trained pediatric radiologist can be potential source of bias. Forty patients meeting the inclusion criteria could not be taken due to non-affordability of the investigations by patients. Potential biases are selection bias as patients reporting in OPD are only included and reporting bias which is due to lack of standardization of lab techniques.

In neurology outpatient ward detailed history and clinical examination was done for each child. Investigations were done based on the suspected diagnosis. Karyotyping and genetic testing were done in patients with family history of GDD and dysmorphism. Metabolic test of blood and urine ketones were done in patients suspected to have metabolic diseases. Electroencephalogram (EEG), visual evoked potential (VEP) and brain stem auditory evoked potential (BAEP) were done if the children had seizure, visual or hearing defect respectively. Neuroimaging (CT and/ or MRI brain) was performed in all affording patients to determine structural abnormality and rule out conditions like neurodegenerative disease of brain. Associated clinical features with GDD and rate of positivity of investigation were also addressed. Collected data was analysed using SPSS and presented in tables. Descriptive statistical analysis was done.

## RESULTS

Over the period of five months, 110 patients fulfilled the criteria of global developmental delay out of which 70 were male and 40 were female. The mean age of the participant at the time of evaluation was 12 months. The etiology was determined in 86 (78%) children

(Table 1).

The most common etiology was perinatal asphyxia 49 (44.5%). The second etiology accounting for GDD was infection 11 (10%) which included six cases of meningitic sequelae, two cases of acute encephalitic sequelae, two congenital rubella syndrome and one cytomegalovirus infection. Similarly there were six cases of genetic syndrome which included four cases of Down's syndrome, one Prader Willi syndrome and one Joubert syndrome. There were six cases of cerebral dysgenesis or neuronal migration disorder which included two cases of schizencephaly, two cases of holoprosencephaly, one corpus callosum agenesis and one Dandy Walker anomaly. There were four cases of neurocutaneous syndrome, two tuberous sclerosis and two Sturge Weber syndrome. GDD of three children was attributed to non-specific leukodystrophy changes.

**Table 1.** Etiology of GDD.

Etiology	Gender Male n (%)	Female n (%)	n (%)
Perinatal asphyxia	36 (73.4)	13 (26.5)	49 (44.5)
Cerebral dysgenesis	2 (33.3)	4 (66.6)	6 (5.45)
Genetic syndrome	3 (50)	3 (50)	6 (5.45)
Metabolic	4 (80)	1 (20)	5 (4.54)
Neurocutaneous	1 (25)	3 (75)	4 (3.63)
Infections	8 (72.7)	3 (27.2)	11 (10)
Nonspecific leukodystrophy like changes	2 (40)	3 (60)	5 (4.54)
Unknown	14 (58.3)	10 (41.6)	24 (21.8)
Total	70 (63)	40 (37)	110

**Table 2.** Associated features with GDD.

Clinical features	n (%)
Abnormal neurological findings	80 (72.7)
Microcephaly	70 (63)
Seizures	49 (44.5)
Dysmorphic	40 (36.3)
Positive family history	18 (16.3)
Autistic features	4 (3.6)

Various factors associated with GDD were analyzed as well which showed abnormal neurological finding in 80 (72.7%) of the patient, followed by microcephaly, seizures and dysmorphic features (Table 2).

**Table 3.** Investigation done and their results.

Investigation	Total (%)	Percentage patients in whom investigation was positive n (%)
MRI	79 (72)	72 (66)
CT	33 (30)	7 (7.7)
EEG	69 (63)	44 (40)
BAEP	62 (57.2)	37 (34)
VEP	50 (46)	30 (28)
TFT	29 (27.2)	11 (10)
Karyotyping	9 (8.1)	7 (6.3)
Metabolic	6 (5.4)	4 (3.6)

MRI was done in 79 (72%) patients out of which 72 (66%) were abnormal. CT was abnormal in 17% of patients (Table 3). The abnormalities found in neuroimaging were encephalomalacia, volume loss, decrease myelination and structure malformation. Metabolic tests showed abnormality in 4 (3.63%) of patients. Karyotyping was found to be abnormal in 7 (6.36%) patients. Among the neurophysiology investigation EEG was abnormal in 69 (63%).

## DISCUSSION

A plethora of etiologies can be attributed for the case of developmental delay.^4^ In children evaluation requires meticulous history taking, examination and judicious use of investigations. The etiologies were detected in 78% of the patients which was slightly higher than the other study.

The prospective study conducted in Oman showed yield of 71%.^2^ Similarly in other retrospective study in Turkey etiology was determined in 63%.^1^ The other prospective study done in Canada and Jordan showed etiological yields of 63% and 54.5% respectively.^5, 6^ The higher yield of etiology in this study is due to higher number of referrals to neurology clinic of Kanti Children's Hospital, being the only tertiary pediatric hospital in the country.

Perinatal asphyxia was the leading etiology in determination of GDD. The burden of perinatal asphyxia is higher in developing countries and associated with poor neurodevelopmental outcome.^7^ The increased perinatal asphyxia can be attributed to difficult geographic conditions, poor antenatal and postnatal care. The rate of the institutional deliveries in our country is only 57% which increases the number of perinatal asphyxia.^8^ Snevall et al. found perinatal asphyxia in 10% of patients.^9^ Prospective study done by Adikari et al in infant of hypoxic ischemic encephalopathy have shown delay in all sectors of development.^10^

Infections contributed to 10% of causes which is higher than study done by Ozman et al. showing 2% of cases of GDD are due to infections.^5^ This shows higher prevalence of infective diseases and their sequelae in our part of world.

Cerebral dysgenesis was an important finding in MRI accounting for 6% of the causes. Koul et al. found it responsible in 12 %^2^ of patients and 10% in study done in Canada.^5^ The lower yield may be because of cost of Neuroimaging that patients can't afford.

Since 2006 AAP published several reports on use of metabolic testing in GDD. ^11^ The metabolic yield in this study was 5% while study in Oman it was 10%. ^1^ The low yield of such testing especially on routine basis has been noted in other studies also.^5^ Metabolic work up should be reserved for the conditions like positive family history, parental consanguinity or developmental regression.

Genetic syndromes were detected in 5.45% of the patients. With recent advances in the yield of cytogentics, genetic study is recommended if etiology is not apparent even after history and examination. Higher yield is found where there is subtle dysmorphism. ^12^

With advances in new technology, more genetic tests allow better elucidation of the etiological diagnosis. Microcephaly was found in 63% patients which is quite similar to 70% in Koulet.al. ^1^ Abnormal neurological finding was important finding associated with 63% of the patients with developmental delay which is similar to 76.4% in Koul et al. ^1^ Dysmorphic features were present in 36.3% of the patients predictive of genetic or chromosomal anomalies. ^2^ However there are reports that dysmorphic features do not predict etiology. The conflict between the studies may be because of subjective judgement and difference in the number of the patient studied.

Neuroimaging was most frequently obtained investigation in studied population. Studies have reported 9% to 80% abnormalities in neuroimaging. ^2^ In this study MRI was positive in 66% and CT in 16% which is same as above range. The yield of MRI is higher in GDD where it is associated with clinical signs such as abnormal head circumference, focal neurological sign or epilepsy. Therefore target use is advocated. MRI is more sensitive test and has no radiation exposure making it preferred choice over CT.^12^

EEG is found abnormal in 40% of the patients which can be useful both for diagnostic and treatment purposes. Particular patterns of EEG are observed in some genetic/chromosomal disorders which contribute to the understanding of pathophysiology and diagnosis. ^1^

This study has certain limitations. The investigations done in this study largely depended on affordability and availability of the tests which has limited the process of finding etiology. Further this study has taken in account of the patients coming to neurology out patient service over five months which is not sufficient time to study varied causes of GDD. CONCLUSIONS

Establishing underlying cause is very important in GDD to identify treatable cause, define recurrence risk and deciding treatment plans. Despite the new advances in technology particularly in realm of genetic investigations, complex MRI protocols, the clinical assessment continues to be very vital in deciding investigations. The investigations like neuroimaging cytogenetics and neurophysiological studies are of great help to establish etiology. Hence they are recommended to use in association with good history and examination.

## Conflict of Interest

**None.**
